# α7 and β2 Nicotinic Acetylcholine Receptor Subunits Form Heteromeric Receptor Complexes that Are Expressed in the Human Cortex and Display Distinct Pharmacological Properties

**DOI:** 10.1371/journal.pone.0130572

**Published:** 2015-06-18

**Authors:** Morten Skøtt Thomsen, Ruud Zwart, Daniel Ursu, Majbrit Myrup Jensen, Lars Hageman Pinborg, Gary Gilmour, Jie Wu, Emanuele Sher, Jens Damsgaard Mikkelsen

**Affiliations:** 1 Neurobiology Research Unit, University Hospital Copenhagen, Rigshospitalet, Copenhagen, Denmark; 2 Lilly Research Centre, Eli Lilly and Company Limited, Erl Wood Manor, United Kingdom; 3 Epilepsy Clinic, University Hospital Copenhagen, Rigshospitalet, Copenhagen, Denmark; 4 Divisions of Neurology, Barrow Neurological Institute, St. Joseph's Hospital and Medical Center, Phoenix, United States of America; Neuroscience Campus Amsterdam, VU University, NETHERLANDS

## Abstract

The existence of α7β2 nicotinic acetylcholine receptors (nAChRs) has recently been demonstrated in both the rodent and human brain. Since α7-containing nAChRs are promising drug targets for schizophrenia and Alzheimer’s disease, it is critical to determine whether α7β2 nAChRs are present in the human brain, in which brain areas, and whether they differ functionally from α7 nAChR homomers. We used α-bungarotoxin to affinity purify α7-containing nAChRs from surgically excised human temporal cortex, and found that α7 subunits co-purify with β2 subunits, indicating the presence of α7β2 nAChRs in the human brain. We validated these results by demonstrating co-purification of β2 from wild-type, but not α7 or β2 knock-out mice. The pharmacology and kinetics of human α7β2 nAChRs differed significantly from that of α7 homomers in response to nAChR agonists when expressed in *Xenopus* oocytes and HEK293 cells. Notably, α7β2 heteromers expressed in HEK293 cells display markedly slower rise and decay phases. These results demonstrate that α7 subunits in the human brain form heteromeric complexes with β2 subunits, and that human α7β2 nAChR heteromers respond to nAChR agonists with a unique pharmacology and kinetic profile. α7β2 nAChRs thus represent an alternative mechanism for the reported clinical efficacy of α7 nAChR ligands.

## Introduction

Neuronal nicotinic acetylcholine receptors (nAChRs) are pentameric ligand-gated ion channels. A total of 11 nAChR subunits (α2–7, α9–10, and β2–4) have been cloned from mammalian neuronal tissue [1]. Of these, the α7 and α9 subunits can form homomeric receptors when expressed in heterologous expression systems, whereas the others assemble into heteromeric structures containing various combinations of α and β subunits [2]. Different combinations of subunits yield nAChRs that differ considerably in their functional and pharmacological properties [1].

With regard to the α7 nAChR, several studies have reported cognitive deficits in α7 knock-out mice and procognitive effects of selective α7 nAChR agonists [3–5]. Further, genetic studies have implicated the *CHRNA7* gene in schizophrenia and Alzheimer’s disease [6–10]. The α7 nAChR is therefore considered a promising drug target for the treatment of cognitive symptoms in patients with schizophrenia or Alzheimer’s disease, and this has led to an intensive drug development effort to produce α7 nAChR selective agonists and positive allosteric modulators (reviewed in [11]).

This research is based on an assumption that the α7-containing nAChRs in the brain are homomers [4,12,13]. However, α7 subunits have been demonstrated to co-assemble with β2, β3, and β4 subunits, respectively, after heterologous expression in *Xenopus* oocytes, and the resulting heteromers are functionally different from α7 nAChR homomers [14–19]. Furthermore, α7 and β2 subunits have been shown to co-immunoprecipitate from rodent tissue from the hippocampus and ventral diagonal band of Broca, but not from the ventral tegmental area [16,20], suggesting that α7β2 nAChR heteromers are present and have a discrete distribution in the mammalian brain. Furthermore Moretti et al. recently demonstrated that α7 and β2 nAChR subunits form complexes in post mortem samples of human basal forebrain [21]. Interestingly, in contrast to the homomeric receptor, heterologously expressed α7β2 nAChR heteromers are highly sensitive to blockade by Aβ_1–42_, suggesting that α7β2 nAChRs may play a unique role in the neuropathology of Alzheimer’s disease [16].

These studies suggest that the function and pharmacology of α7-containing nAChRs in the brain may be more diverse than has previously been appreciated, and that the function of native α7 nAChRs may depend on multiple interaction partners [22]. Notably, the potential presence of α7β2 nAChR heteromers in the human cortex may have a significant impact on the clinical efficacy of ligands targeting the α7 subunit to improve cognition.

The first aim of this study was therefore to determine if α7 and β2 subunits coassemble in the human cortex using affinity purification with the highly selective α7 antagonist α-bungarotoxin (α-Bgt). Next, we studied potential differences in pharmacology and kinetics of α7β2 compared to α7 nAChRs in *Xenopus* oocytes and HEK293 cells in response to several α7 nAChR ligands.

## Experimental procedures

### Tissue

Human temporal neocortical tissue was obtained from anterior temporal resections performed in patients with medically intractable temporal lobe epilepsy with hippocampal onset. The study was approved by the Ethical Committee in the Capital Region of Denmark (H-2-2011-104) and performed in accordance with the Declaration of Helsinki. Written informed consent was obtained from both patients before surgery.

The tissue was dissected and immediately frozen on dry ice and stored at -80°C until use. The tissue contained all layers of the neocortex included the underlying white matter. Two separate specimens were used for the present studies (females aged 30 and 44). The neuropathological examinations of the neocortex from both patients were normal.

Mice deficient for the α7 or β2 nicotinic receptor (C57BL/6 background) or KO, and wild type (WT) littermates were purchased from The Jackson Laboratories and bred at Virginia Commonwealth University. 8–12 weeks old male or female homozygous knockout and age- and sex-matched wild-type mice were sacrificed by decapitation and the brains dissected and stored at -80°C. Cortical tissue was dissected using a scalpel.

### Chemicals

All chemicals to prepare the recording and Barth’s solutions, as well as dimethyl sulfoxide, acetylcholine chloride (ACh), carbamylcholine chloride (carbachol) and choline chloride were obtained from Sigma-Aldrich (Poole, U.K.). Dihydro-β-erythroidine hydrobromide (DHβE), methyllycaconitine citrate (MLA), (±)-epibatidine, and PNU120596 were purchased from Tocris (Bristol, U.K.). Compound B ([R-N-(1-azabicyclo[2,2,2]oct-3-yl)(2-pyridyl)thiophene-2-carboxamide, previously also named as compound A) was synthesized at Eli Lilly & Co and had a purity greater than 95% [19].

### Affinity purification using α-bungarotoxin

α-Bgt (Biotium, Hayward, CA) was dissolved to 2.22 mg/ml in PBS, pH 7.4 at 4°C for 72 hours after which it was coupled to PureProteome NHS Flexibind magnetic beads (Millipore, Billerica, MA) in a ratio of 1:1.6 (vol/vol) using the manufacturer’s instructions. Successful coupling was confirmed by subsequent protein determination showing a substantial decrease in the protein content of the α-Bgt solution. Another batch of beads was processed in parallel, but with no α-Bgt in the PBS, as a negative control. The beads were incubated in 0.1% bovine serum albumin in PBS, pH 7.4 for 1 hour at 4°C prior to use.

The tissue was lysed in 1 ml lysis buffer (50 mM Tris, 50 mM NaCl, 5 mM EDTA, 5 mM EGTA, 10 μl/ml protease inhibitor cocktail (Sigma-Aldrich, Brøndby, Denmark), pH 7.5) using a PT1200C polytron blender (Kinematica, Luzern, Switzerland) for 20 seconds. The lysate was centrifuged 30 minutes at 160,000 × g at 20–22°C using an air-driven ultracentrifuge (Airfuge, Copenhagen, Denmark), and the supernatant was discarded. The pellet was resuspended in 1 ml lysis buffer containing 2% Triton X-100 by blending for 20 seconds, and incubated for 2 hours at 4°C on a rotor (15 rpm). Thereafter, the sample was centrifuged as above and the resulting supernatant used for affinity purification. Total protein content was determined using the Pierce 660nm Protein Assay (Thermo scientific, Rockford, IL) and 500–800 μg protein was incubated with 50 μl magnetic beads in a total volume of 1500 μl lysis buffer for 18–22 hours at 4°C on a rotor (15 rpm). When comparing affinity purification with homogenates from α7^+/+^ and a mixture of α7^-/-^ and β2^-/-^ mice, the mixture was 1:1 and had double the amount of total protein in order to have equal amounts of α7 and β2 subunits, Subsequently, the beads were washed twice in 1 M NaCl, 8 mM Na_2_HPO_4,_ 2 mM NaH_2_PO4, 0.5% Triton X-100, pH 7.5 and three times in 0.1 M NaCl, 8 mM Na_2_HPO_4,_ 2 mM NaH_2_PO4, 0.5% Triton X-100, pH 7.5 and immediately processed for western blotting.

### Western blotting

Samples were diluted in loading buffer (final concentration: 60 mM Tris, 10% (v/v) glycerol, 5% (v/v) mercaptoethanol, 2% (w/v) SDS, 0.025% (w/v) bromophenol blue, pH 6.8), incubated for 5 minutes at 95°C and submitted to gel electrophoresis using AnykD gels (Biorad, Hercules, CA), and blotted onto PVDF membranes (BioRad). Membranes were washed in TBS-T and blocked in TBS containing 5% (w/v) dry milk powder, which was also used for antibody incubations. Incubation in primary antibody against α4 (1:100, sc-5591, Santa Cruz Biotechnology, Heidelberg, Germany), α7 (1:1000, ab23832, Abcam, Cambridge, UK), or β2 (1:1,000, a gift from Dr. Cecilia Gotti, which we have characterized previously [23]) was performed overnight at 4°C on parafilm in a humidified container, followed by 3 × 10 minute washes in TBS-T and 1 hour incubation at 20–22°C in horseradish peroxidase-coupled secondary antibody (1:1,000, Dako, Glostrup, Denmark). After thorough washing in TBS-T, enhanced chemiluminescence Western blotting detection reagents (Western Lightning ECL Pro, Perkin Elmer, Waltham, MA) were used for signal detection and protein bands were visualized using a Chemidoc XRS system with Quantity One software (Biorad). Mean optical densities of bands were measured and their corresponding background measurement subtracted.

### nAChR expression and two-electrode voltage clamp on Xenopus oocytes

*Xenopus* oocyte expression and electrophysiological recordings were performed as described before [24]. Briefly, stage V and VI *Xenopus* oocytes (Xenopus Express, Vernassal, France) were prepared using standard procedures. Human α7 and β2 subunit cDNAs, ligated into the pcDNA3 (Invitrogen) expression vector, were dissolved in distilled water at a concentration of 0.1 mg/ml (spectrophotometric determinations). α7 cDNA or a mixture of α7 and β2 cDNA at a 1:10 ratio was injected into the nuclei of oocytes in a volume of 18.2 nl/oocyte, using a Nanoject Automatic Oocyte Injector (Drummond, Broomall, PA). The total amount of cDNA injected per oocyte was kept constant at 2 ng. After injection, oocytes were incubated at 18°C for 3–5 days in a modified Barth’s solution containing 88 mM NaCl, 1 mM KCl, 2.4 mM NaHCO_3_, 0.3 mM Ca(NO_3_)_2_, 0.41 mM CaCl_2_, 0.82 mM MgSO_4_, 15 mM Hepes and 5 mg/l neomycin, pH 7.6. Recordings were performed 3–5 days post-injection. Oocytes were placed in a 0.1 ml recording chamber and perfused with a recording solution (containing in mM: NaCl 150, KCl 2.8, Hepes 10, CaCl_2_ 1.8; MgCl_2_ 1.0; pH 7.2, adjusted with NaOH) at a rate of 10 ml/min.

Oocytes were impaled by two microelectrodes filled with 3 M KCl (0.5–2.0 MΩ) and voltage-clamped at –60 mV using a Geneclamp 500B amplifier and PCLAMP 6 software (Axon Instruments, CA, U.S.A.). Typically, traces were filtered at 100 Hz during recording and digitized at 500 Hz using the DigiData 1200 interface (Axon Instruments, CA). All experiments were carried out at room temperature. Agonist concentration-response curves were obtained by normalizing agonist-induced responses to control responses induced by 1 mM ACh (a near-maximum effective concentration at α7 as well as α7β2 receptors). An interval of 2 minutes was allowed between agonist applications, as this was found to be sufficient to ensure reproducible recordings.

Concentration-response curves were fitted by a non-linear least-squares algorithm according to the equation:

$$\text{i}={\text{i}}_{\text{max}}\;/\;\left(1+{\left\{{\text{EC}}_{50}/\left[\text{conc}\right]\right\}}^{\text{n}}\right)$$ (1)

in which i_max_ is the maximum obtainable peak current, EC_50_ is the concentration of the agonist that elicits 50% of the maximum obtainable peak current, and n is the slope factor. Results are expressed as mean ± standard error of the mean.

### nAChR expression and patch clamp on transfected HEK-293 cells

Patch clamp experiments were performed on transiently transfected HEK-293 cells. Transfections were performed in 6 well plates by using Lipofectamine 2000 (Invitrogen, Paisley, UK), and 2 μg α7 or α7β2 (1:10) plasmid, using the same plasmids as in the oocyte experiments. RIC-3 was used in the transfection protocol in a ratio 1:1 with α7 or α7/β2 plasmid, (2 μg) in order to boost trafficking of α7-containing nAChRs. Transfected cells were plated in 35 mm bottom glass dishes the day after transfection and used for whole-cell voltage-clamp recordings after 48h. During recordings, cells were continuously perfused in HEPES-buffered Tyrode’s solution (HBTS, Invitrogen, Paisley, UK) containing (in mM): 135 NaCl, 5 KCl, 1.2 MgCl_2_, 2.5 CaCl_2_, 10 HEPES, 11 glucose (pH = 7.2). Cells were voltage-clamped in the whole-cell configuration at a holding potential of -60 mV with an AxoPatch 200A patch-clamp amplifier (Molecular Devices, Sunnyvale, CA, USA). Pipettes were pulled from borosilicate glass (Type GC150F-10, Harvard Apparatus, Kent, UK) using a commercial puller (Model P-87, Sutter Instruments, Novato, CA, USA) and had resistances between 2 and 4 MΩ when filled with pipette solution containing (in mM): 127 CsCl, 1 MgCl2, 4 MgATP, 10 EGTA, 10 HEPES, 10 NaCl (pH = 7.3). Current data were recorded at 10 kHz using a DA/AD interface (Digidata 1322A, Molecular Devices, Sunnyvale, CA, USA). Drugs were applied using a multichannel perfusion system (Model BPS-8, Scientifica, Uckfield, UK) positioned 150 μM away from the recorded cell and controlled by Clampex 9 software (Molecular Devices, Sunnyvale, CA, USA).

### Single cell calcium imaging

Fluorescence-based calcium imaging experiments were performed as described before [25]. Briefly HEK-293 cells (European Collection of Cell Cultures, #85120602) were transfected using the transfection protocol specified above and plated at a density of 20 x 10^5^ cells/ml (50 μl per well) on poly(D)-lysine/laminin-coated black-walled clear bottom 96-well plates (Corning, UK). Cells were loaded in the dark for 60 minutes at 22°C in HBTS containing 4 μM of the calcium-sensitive dye Fluo-4 AM (Invitrogen, Paisley, UK) in the presence of 1% pluronic acid (Invitrogen). Cells were washed and continually perfused during the experiment with HBTS. Dye-loaded cells were viewed using an inverted epifluorescence microscope (Axiovert,135TV, Zeiss, Cambridge, UK). Fluo-4 fluorescence was excited by a 480±10nm light source (Polychrome II, TILL-Photonics, Gräfelfing, Germany) and emission was captured by a iXon 897 EMCCD camera (Andor Technologies, Belfast, UK) after passage through a dichroic mirror (505LP nm) and a high pass barrier filter (515LP nm). Digitised images were stored and processed using Imaging Workbench 5.0 software (INDEC Biosystems, Santa Clara, CA, USA). Data were analysed by averaging individual traces collected from a large number of cells in multiple wells of the 96-well plate. Delta F/F0 values were measured by quantifying the ratio between the change in fluorescence signal intensity (delta F) and baseline fluorescence (F0).

### Statistical analyses

Unpaired t-tests were used to compare time constants and log EC50 and IC50 values from *Xenopus* oocytes and peak currents in HEK293 cells. The statistical calculations were performed using GraphPad Prism version 6.03 for Windows (GraphPad Software, San Diego, USA). All data are presented as mean±standard error of the mean, and a *P*-value of less than 0.05 was considered statistically significant.

## Results

### α7-containing nAChRs form heteromers with β2 nAChR subunits in mouse cortex

**Fig 1:**
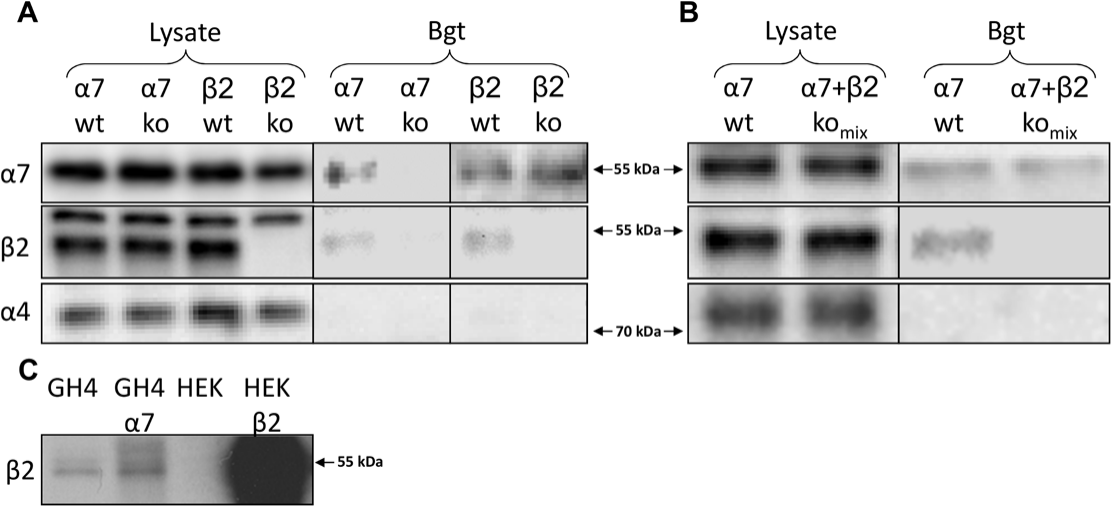
Bead-coupled α-bungarotoxin selectively purifies α7-containing nAChRs from mouse cortex. (A) Affinity purification was performed with bead-coupled α-bungarotoxin (α-Bgt) on cortical homogenates from α7^+/+^, α7^-/-^, β2^+/+^, and β2^-/-^ mice and the isolated proteins were separated using gel electrophoresis. Subsequent detection using Western blotting demonstrated the presence of the α7 and β2 in the isolates from α7^+/+^ and β2^+/+^ mice. In isolates from α7^-/-^ mice none of these proteins were detected, demonstrating that α-Bgt specifically isolates α7-containing nAChRs, and that the detection of β2 is dependent on the presence of α7 protein. In isolates from β2^-/-^ mice, there was no detection of β2 protein, confirming the identity of the band isolated using α-Bgt as being β2. The α4 subunit was not detected in any of the isolates, confirming that the presence of β2 protein is not due to non-specific isolation of α4β2 nAChRs. In the original tissue lysates α7, β2, and α4 protein was readily detectable, except that β2 is not detected in β2^-/-^ lysates, demonstrating the specificity of the antiserum. A 55 kDa protein was detected in both α7^+/+^ and α7^-/-^ lysates, as has previously been shown for several other α7 antibodies [26,27]. (B) α-Bgt affinity purification on cortical homogenates from α7^+/+^ and a mixture of α7^-/-^ and β2^-/-^ mice. β2 subunits were not detected in the latter. (C) Detection of β2 is evident in HEK293 cells transfectd with the human β2 gene (HEK β2), but not in untransfected cells (HEK). Similarly transfection of GH4 cells with human α7 (GH4 α7) does not alter detection of the band corresponding to β2.

We applied affinity purification using bead-coupled α-Bgt followed by Western blotting and antibody detection to detect α7-containing nAChRs from extracts of mouse cortex., After isolation using bead-coupled α-Bgt, a 55 kDa protein band was readily detected from cortical homogenates from α7^+/+^, but not α7^-/-^ mice. The absence of the band in α7^-/-^ mice, clearly indicates that the band corresponds to the α7 protein, and thus demonstrates specific isolation of α7-containing receptors using this approach (Fig 1A). The absence of a band corresponding to α7 protein in α7^-/-^ mice also demonstrated the specificity of the used anti-α7 antibody in α-Bgt affinity purified samples. Without α-Bgt affinity purification, a 55 kDa protein band was readily detected in both α7^+/+^ and α7^-/-^ lysates, as previously shown for other α7 antisera [26,27]. This could be because the antibody used is raised against a part of the protein coded by exons 1–4, which are not deleted in the α7^-/-^ mice, although binding of the antibody to other cellular targets sharing a similar epitope cannot be excluded [28]. Consequently, we were unable to determine the proportion of total α7 protein that was isolated by α-Bgt affinity purification.

We further showed that α7-containing nAChRs isolated from the cortex of α7^+/+^ and β2^+/+^ mice using bead-coupled α-Bgt co-purified with β2 nAChR subunits (Fig 1A). No β2-immunoreactivity was detected in purified extracts from α7^-/-^ mice, confirming that co-purification of β2 only occurred in the presence of α7 protein. Similarly, no β2-immunoreactivity was detected in isolates from β2^-/-^ mice, confirming the identity of the band isolated using α-Bgt wild-type mice as being the β2subunit.

The β2 antiserum immunoreacted with two separate bands around the expected molecular weight, but only the lower band was absent in β2^-/-^ lysates, demonstrating that this band corresponds to the β2 nAChR subunit. This band corresponded to the β2 band observed in α-Bgt affinity purified samples. The α4 nAChR subunit was readily detectable in the original tissue lysates used for affinity purification, but was not detected in any of the isolates, suggesting that the presence of β2 subunits is not due to non-specific isolation of α4β2-containing nAChRs.

Affinity purification performed with a 1:1 mixture of cortical homogenates from α7^-/-^ and β2^-/-^ mice revealed no co-purification of β2 nAChR subunits (Fig 1B).

To confirm that the β2 antibody did not detect the α7 subunit, we demonstrate that transfection of GH4 cells with human α7 does not alter detection of the band corresponding to β2, whereas expression of β2 in HEK cells is readily detectable (Fig 1C).

### α7-containing nAChRs form heteromers with β2 nAChR subunits in human cortex

**Fig 2:**
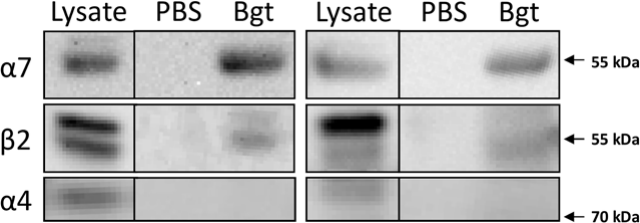
α7-containing nAChRs form heteromers with β2 nAChR subunits in human cortex. Magnetic beads covalently coupled with α-bungarotoxin (α-Bgt) or uncoupled beads (PBS) were incubated with homogenates from human temporal cortex (two separate identical experiments are shown) and the isolated proteins were separated using gel electrophoresis. Subsequent detection Western blotting demonstrated the presence of the α7 and β2 from samples isolated using α-Bgt-coupled, but not uncoupled beads. The α4 subunit was not detected in any of the isolates, confirming that the presence of β2 protein is not due to non-specific isolation of α4β2 nAChRs.

We used the same affinity purification method to purify α7-containing receptors from two human temporal cortex biopsies (Fig 2), and we were able to show co-purification of β2 nAChR subunits with α7 in extracts from both human cortical biopsies. No bands were detected when performing the affinity purification with uncoupled beads, confirming that the isolation of α7 and β2 was attributable to a specific interaction with α-Bgt.

No immunoreactivity for the α4 subunit was detected in any of the isolates, confirming that the presence of β2 subunits is not due to non-specific isolation of α4β2-containing nAChRs.

### α7β2 nAChRs display different pharmacology from α7 nAChRs in Xenopus oocytes

**Fig 3:**
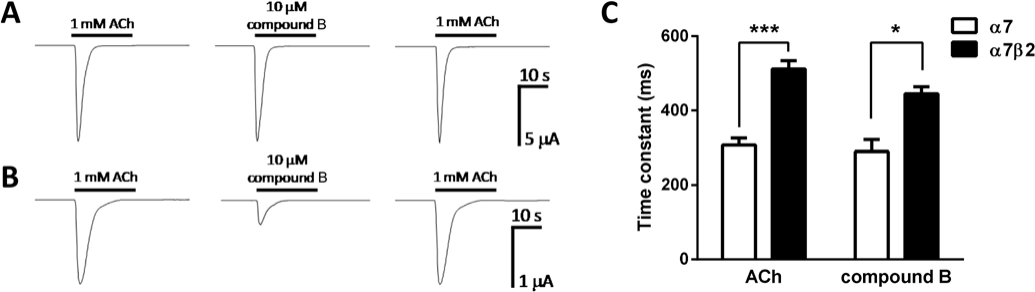
Responses of human α7 and α7β2 nAChRs to the nAChR agonists acetylcholine (ACh) and compound B in *Xenopus* oocytes. A) 1 mM ACh and 100 mM compound B evoke rapidly activating and desensitizing inward currents in oocytes expressing human α7 nAChRs. B) 1 mM ACh and 100 μM compound B evoke rapidly activating and desensitizing inward currents in oocytes expressing human α7β2 (1:10) nAChRs. C) Time constants for the decay phases of the responses to ACh (n = 6) and compound B (n = 3) for α7 and α7β2 nAChRs when fitted to a mono-exponential function. *** *P*<0.001 and * *P*<0.05 indicates significant difference in an unpaired t-test.

When human α7 nAChR cDNA was expressed in oocytes, rapid activation and desensitization within about a second back to baseline was observed upon application of 1 mM ACh. Similarly, the selective α7 nAChR partial agonist compound B [25] at 100 μM evoked a rapidly activated inward current that also completely desensitized back to baseline (Fig 3A). In oocytes expressing both α7 and β2 nAChR subunits, 1 mM ACh induced a rapidly activating inward current that desensitized back to baseline, but the peak current responses were smaller and the speed of desensitization was slower than that of α7 homomers (Fig 3B). Compound B at 100 μM also induced a rapidly activating inward current, but again the α7β2 nAChR-mediated response were smaller and desensitized slower than that of α7 homomers. When fitted to a mono-exponential function, time constants for the decay phases of the responses to 1 mM ACh and 100 μM compound B were significantly slower for α7β2 compared to α7 nAChRs (*P*<0.001 and *P*<0.05, respectively, Fig 3C. The time constants of desensitization for both nAChR subtypes did not differ significantly depending on the agonist used.

**Fig 4:**
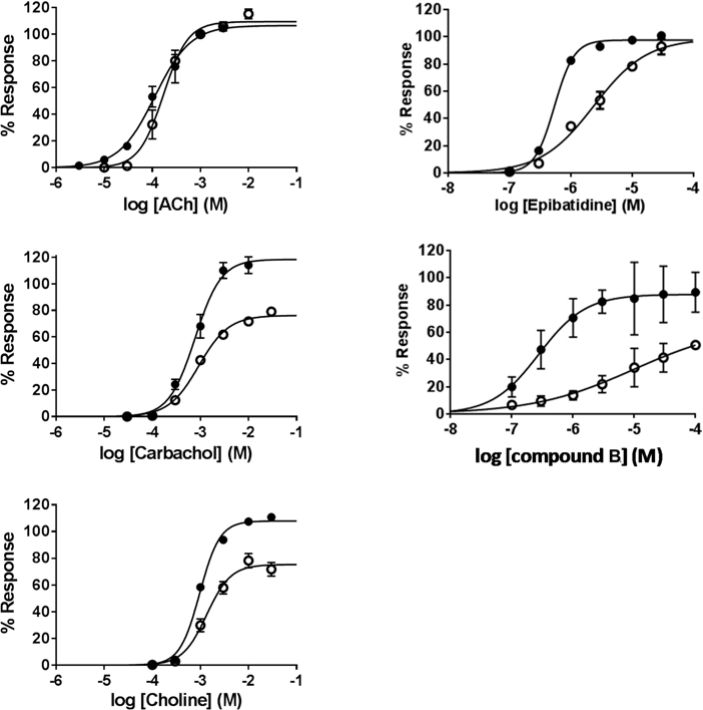
Concentration-response curves of nAChR agonists on human α7 and α7β2 nAChRs in *Xenopus* oocytes. Acetylcholine (ACh), carbachol, choline, epibatidine and compound B were applied in various concentrations to *Xenopus* oocytes expressing α7 (filled circles) and α7β2 (1:10, empty circles) nAChRs. All responses were normalized to the peak amplitude of a 1 mM control ACh-induced ion current in the respective oocyte.

Concentration-response curves for the two human receptors were measured for the agonists ACh, carbachol, choline, epibatidine, and compound B (Fig 4). Each concentration of agonist was applied for 2 seconds and peak current amplitudes were normalized to the average peak amplitude of a control 1 mM ACh induced current that were evoked before and after each concentration of test compound. Fitting the normalized responses to the Hill equation revealed that carbachol, choline and compound B had lower Emax in α7β2 nAChRs compared to α7 nAChRs, and that epibatidine and compound B had significantly lower EC_50_. Estimated parameters for EC_50_, Emax, and slope factor are summarized in Table 1.

**Table 1:** Summary of agonist and antagonist concentration-response curves on human α7 and α7β2 nAChRs expressed in *Xenopus* oocytes.

		hα7				hα7β2 (1:10)			
**Agonist**	**EC50 (μM)**	**Emax (%)**	**nH**	**n**	**EC50 (μM)**	**Emax (%)**	**nH**	**n**	**p**
**ACh**	113 (78–163)	106 ± 6	1.17 ± 0.18	3	171 (138–211)	109 ±3	1.70 ± 0.25	3	0.19
**Carbachol**	779 (598–1013)	118 ± 5	1.60 ± 0.25	3	923 (781–1090)	76 ± 2	1.39 ± 0.13	5	0.34
**Choline**	972 (888–1064)	108 ± 2	2.09 ± 0.19	3	1350 (1039–1755)	75 ± 4	1.74 ± 0.32	4	0.18
**Epibatidine**	0.54 (0.25–0.89)	98 ± 1	2.70 ± 0.13	4	(1.5–3.8)	99 ± 7	1.01 ± 0.15	3	0.046*
**Comp. B**	0.28 (0.15–0.51)	88 ± 5	1.14 ± 0.39	6	9.8 (4.9–19.6)	65 ± 21	0.53 ± 0.18	6	0.039*
**Antagonist**	**IC50 (**μ**M)**		**nH**	**n**	2.4 **IC50 (**μ**M)**		**nH**	**n**	
**DHβE**	47 (41–53)		2.74 ± 0.31	3	18 (14–23)		1.09 ± 0.14	3	0.003**
**MLA**	0.00089 (0.00074–0.00108)		1.69 ± 0.20	3	0.0013 (0.0011–0.0015)		1.60 ± 0.19	3	0.044*

All responses were normalized to the peak amplitude of a 1 mM control ACh-induced ion current. Antagonists were first pre-applied for 1 min followed by co-application with 1 mM ACh. During curve fitting of agonist data the bottom of the curves were constrained to 0 and curve fitting of antagonist data was performed by constraining the top and the bottom of the curves at 100 and 0%, respectively. T-tests on EC50 and IC50 data was performed on log EC50 and log IC50 values. Statistical significance

* *P*<0.05

** *P*<0.005.

**Fig 5:**
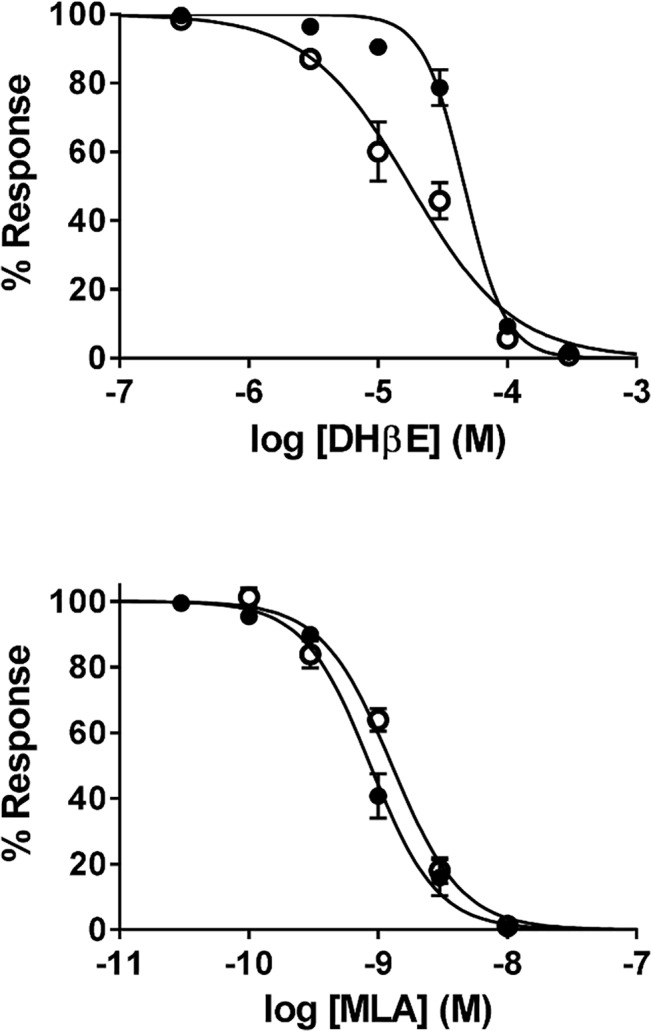
Effect of antagonists on human α7 and α7β2 nAChRs in *Xenopus* oocytes. Inhibition curves for dihydro-β-erythroidine (DhβE) and methyllycaconitine (MLA) on α7 (filled circles) and α7β2 (1:10, empty circles) nAChRs. Co-expression of α7β2 nAChR subunits lead to a significantly decreased IC_50_ for DhβE (*P*<0.01), and an increased IC_50_ for MLA (*P*<0.05) compared to α7 nAChR homomers.

Similarly, concentration-response curves were also obtained for the antagonists DHβE and MLA (Fig 5). Various concentrations of antagonist were pre-applied for 1 min upon which it was co-applied with 1 mM ACh. Responses to 1 mM ACh in the presence of antagonist were normalized to the average of the 1 mM ACh-induced responses that were evoked before and after each antagonist application. Fitting the normalized responses to the Hill equation revealed that DHβE had significantly higher while MLA had significantly lower potency for α7β2 compared to α7 nAChRs (*P*<0.01 and *P*<0.05, respectively).

**Fig 6:**
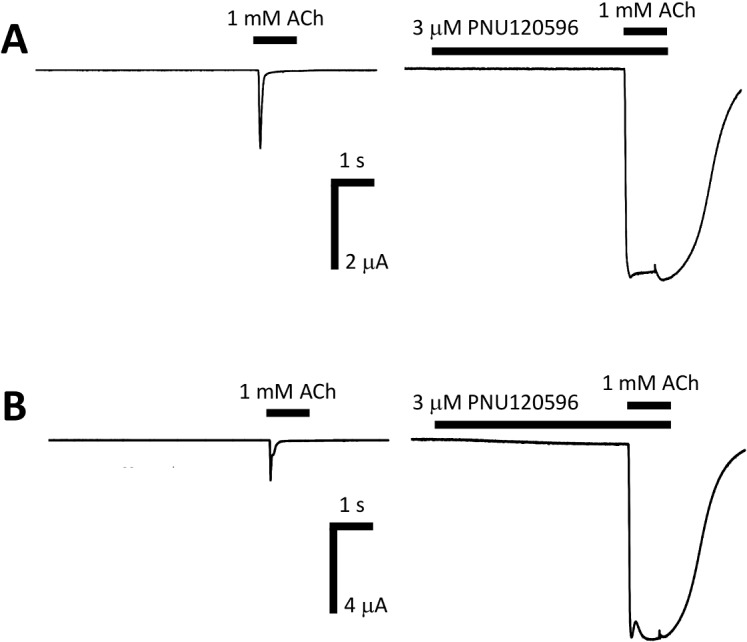
Potentiation of human α7 and α7eβ2 nAChRs in Xenopus oocytes by the allosteric potentiator PNU120596. A) A control response was evoked by applying 1 mM ACh to an α7 nAChR expressing oocyte. After a 3 min wash period 3 μM PNU120596 was applied to the same oocyte and after 1 min PNU120596 was co-applied with 1 mM ACh. In the presence of PNU120596 the peak of the 1 mM ACh-induced response was largely potentiated. B) A control response was evoked by applying 1 mM ACh to an α7β2 nAChR expressing oocyte. After a 3 min wash period 3 μM PNU120596 was applied to the same oocyte and after 1 min PNU120596 was co-applied with 1 mM ACh. In the presence of PNU120596 the peak of the 1 mM ACh response was largely potentiated.

One minute pre-application with 3 μM of the α7 nAChR positive allosteric modulator PNU120596 resulted in a large potentiation of 1 mM ACh-induced response in an oocyte expressing hα7 nAChRs (2.5 ± 0.9 fold; n = 3, Fig 6A). 3 μM PNU120596 also potentiated human α7β2 nAChRs to a large extent (4.7 ± 2.8 fold; n = 3, Fig 6B), but this was not significantly different from the response to hα7 nAChRs.

### α7β2 nAChRs display different pharmacology from α7 nAChRs in HEK293 cells

**Fig 7:**
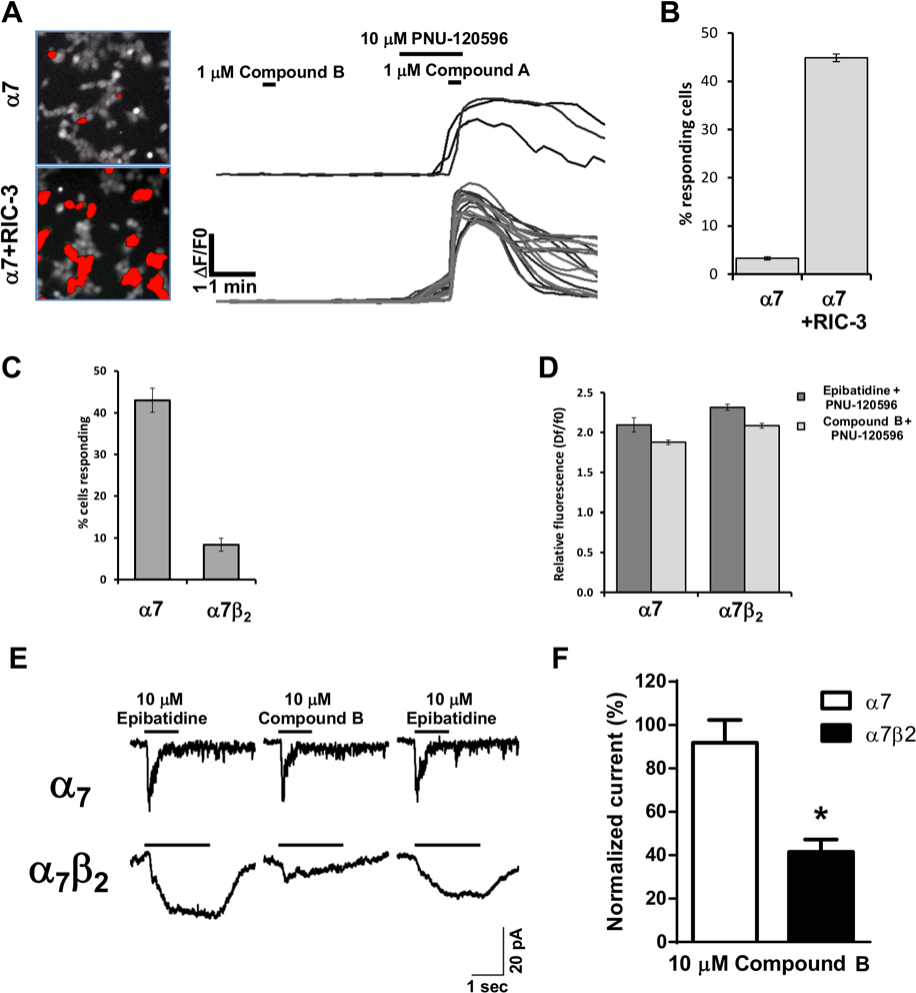
Responses of human α7 and α7β2 nAChRs to the nAChR agonists epibatidine and compound B and the modulator PNU120596 in HEK293 cells. A) Efficiency of α7 expression was determined as the number of regions of interest (outlined in red in the left panel images) showing a fluorescence increase above a threshold of 0.1 ΔF/F0 following application of compound B and PNU-120596. Representative single cell traces are shown for the two transfection conditions with α7 alone and the combination of α7 and RIC-3. B) Relative expression efficiency for the experiment described in A. C) Expression efficiency in HEK293 cells transfected with either α7 or α7β2 cDNA in the presence of RIC-3. D) The corresponding relative response amplitude for the two types of transfected cells obtained following co-application of either epibatidine (10 uM) or compound B (1 uM) and the α7 positive allosteric modulator PNU-120596 (10 uM). For data presented in panels A-D n = 3 for each experimental condition. E) Representative patch clamp recordings showing responses to the application of either epibatidine (10 M) or compound B (10 M) to cells expressing α7 (top traces) or α7β2 (bottom trace) receptors. F) Averaged normalized data for the traces shown in E corresponding to 3 cells (out of 8 recorded for the α7 and 13 recorded for the α7β2 transfected cells) that responded to the agonist application and were considered for analysis. Peak currents corresponding to application of compound B were normalised to the 1^st^ epibatidine application. * *P*<0.05 indicates significant difference in an unpaired t-test.

Expression of α7 nAChRs in HEK293 cells was initially shown by using single cell calcium imaging responses to co-application of compound B and the α7 selective positive allosteric modulator PNU120596. (Fig 7A and 7B). As shown previously [29] co-expression of α7 with RIC-3 greatly enhanced the efficiency of the expression, as shown in our experiment by the large increase in the number of responding cells (44.9±0.8% in the presence vs 3.3±0.3% in the absence of RIC-3 respectively). Therefore, all subsequent experiments used RIC-3 as a co-expression cDNA. The amplitude of the agonist/modulator combination was very large while application of the compound B alone did not elicit any calcium fluxes. This is in agreement with previous studies [25] that showed that the transient nature of the α7 nAChR response is not sufficient to generate calcium fluxes that can be detected in this type of assay. We then compared directly the expression and relative response amplitudes of the α7 and α7β2 (Fig 7C and 7D). Co-expression of α7 and β2 caused a significant reduction in the number of responding cells (from 43.0±3.9% to 8.4±1.6%). The reduction in expression efficacy was not caused by a decrease in the amplitude of responses induced by epibatidine (2.09±0.09 and 2.32±0.03 dF/F0 for α7 and α7β2, respectively) or compound B (1.88±0.04 and 2.08±0.03 dF/F0, respectively) in the presence of the potentiator (Fig 7D).

When expressed in HEK293 cells α7 nAChRs rapidly activated upon application of 10 μM epibatidine and desensitized within about a second back to baseline. The selective α7 nAChR agonist compound B at 10 μM evoked a similar rapidly activated inward current that also completely desensitized to baseline (Fig 7E). In HEK293 cells expressing α7β2 nAChRs 10 μM epibatidine induced a slowly activating inward current that reached a plateau before desensitizing. Compound B at 10 μM induced a very small slowly activating current with a very slow desensitization. Analysis of the peak currents normalized to the response to 10 μM epibatidine, revealed a much reduced current in response to compound B in α7β2 compared with α7 transfected cells (*P*<0.05, Fig 7F).

## Discussion

We demonstrate that native human cortical α7-containing nAChRs can form a complex with β2 nAChR subunits. This is the first evidence for the presence of native α7β2 nAChR heteromers in the human cortex. We further show that human α7 and α7β2 nAChRs display marked pharmacological and kinetic differences in heterologous expression systems.

Previous studies have shown that α7 and β2 nAChR subunits co-immunoprecipitate from the rat and mouse brain [16,20]. However, these studies employed α7 and β2 antibodies that have been shown to stain knock-out tissue [26,27], which demands skepticism regarding co-immunoprecipitation results obtained using such antibodies. We used affinity purification with the highly selective α7 nAChR antagonist α-Bgt to determine that α7 and β2 nAChR subunits co-purify from mouse cortex. We validated the method by showing that genetic ablation of either α7 or β2 abolished this co-purification and by showing that α4 subunits were not co-purified, indicating that the co-purification of β2 protein was not due to non-specific purification of α4β2 nAChRs. To confirm that the α7β2 complex occurred *in vivo*, we show that α-Bgt affinity purification on an extract containing tissue from both α7^-/-^ and β2^-/-^ mice does not yield co-purification of β2 subunits. We further demonstrated that the antibodies used for detection of α7 and β2 nAChR subunits were specific under the conditions used.

Importantly, we were also able to demonstrate co-purification of α7 and β2 nAChR subunits from human temporal cortex, indicating that native α7β2-containing nAChR heteromers are present in the human cortex. This finding complements a recent study demonstrating the existence of α7β2-containing nAChR heteromers in the human basal forebrain [21], and since the basal forebrain projects widely to the cortical mantle [30], this suggests that at least part of the α7β2 nAChRs in the cortex might stem from cell bodies in the basal forebrain. Since α-Bgt binds to the interface between α7 subunits [31], we cannot use α-Bgt to detect protein from nAChR subunits that do not have α7-α7 interfaces, i.e. receptors that contain one or two α7 subunits which are not adjacent in the receptor structure. Therefore, we cannot preclude the presence of other α7-containing heteromeric nAChRs in addition to α7β2 nAChRs.

Co-purification of subunits does not prove that the subunits form functional receptors, but the potential presence of functional α7β2 nAChR heteromers in the human brain raises the possibility that compounds developed as selective ligands for α7 nAChR homomers may also affect heteromeric receptors in the human brain. To study the pharmacological consequences of incorporation of β2 subunits into α7-containing nAChRs, electrophysiological recordings were performed with human α7 and β2 subunits expressed in *Xenopus* oocytes and HEK293 cells. In order to compare the present human α7β2 nAChR data with previously reported rat α7β2 nAChR data [15,19], we measured peak-amplitudes of agonist-induced ion currents rather than the net charge to create concentration-response curves. Peak responses and rate of desensitization for α7β2 nAChR heteromers were generally lower than those of homomeric α7 nAChRs, which is in line with results from previous studies expressing mammalian α7β2 nAChRs in oocytes [15,18,19]. Also in line with previous studies, the concentration-response curve for ACh was similar for human α7β2 and α7 nAChRs, whereas the full α7 agonists carbachol and choline were only partial agonists on α7β2 nAChRs.

Furthermore, the selective α7 partial agonist compound B had a lower efficacy at α7β2 compared to α7 nAChRs, whereas the non-selective nAChR ligand epibatidine was a full agonist on both α7β2 and α7 nAChRs. Similar results have been shown with rat α7β2 nAChRs [19], with two notable differences: compound B has much higher efficacy on human compared to rat α7β2 nAChRs, and the concentration-response curves for compound B and epibatidine on human α7β2 nAChRs were less steep than observed with rat α7β2 nAChRs, resulting in an increased EC_50_ [19].

Co-expression of α7β2 nAChR subunits lead to a decreased IC_50_ of the α4β2 preferring antagonist DHβE in *Xenopus* oocytes, which corroborates that β2 subunits are incorporated into α7-containing nAChRs. The IC_50_ of MLA was slightly increased in α7β2 nAChRs, which may be the result of a decreased number of binding sites compared to α7 homomers, due to the incorporation of β2 subunits.

Since expression of nAChRs in *Xenopus* oocytes have several limitations, including the possible formation of different subunit stoichiometries, and a current contribution from calcium-dependent chloride channels [32,33], we also investigated the function of α7 versus α7β2 nAChRs expressed in the mammalian cell line HEK293. In HEK293 cells, epibatidine exhibited similar peak response currents in α7 and α7β2 nAChR expressing cells, which differs from the 5 fold difference seen in *Xenopus* oocytes, whereas α7β2 nAChRs displayed a reduced current response to compound B compared to α7 nAChR homomers. But more strikingly, the profile of the response to epibatidine and compound B was completely different in HEK293 cells compared to *Xenopus* oocytes. The α7β2 nAChRs displayed a slow rise and decay phase, resulting in a very long response time, which was not seen with α7 homomers. These data demonstrate a much more pronounced kinetic difference between α7β2 and α7 nAChRs than is evident when the receptors are expressed in *Xenopus* oocytes [18], and suggest that α7β2 nAChRs in the human brain may display markedly different kinetics from α7 nAChR homomers. Importantly, the slow kinetics observed for α7β2 may facilitate activation of Ca^2+^-dependent intracellular signalling and neuronal excitability [34], suggesting that the α7β2 nAChR may affect neuronal excitability to a much larger degree than α7 nAChR homomers. The number of responding HEK293 cells expressing α7β2 was much lower compared to those expressing α7 nAChRs. This may reflect the fact that they expressed did not have α7-α7 interfaces, which might be required for full activation of α7β2 nAChR [18,21], or it could be due to potential dominant negative effects of β2 presence on the assembly and trafficking of the receptors.

The α7β2-containing nAChRs constitute a particularly interesting molecular target for three main reasons: 1) α7β2 nAChRs are found in the basal forebrain [16] where it has been shown that α7 and β2 mRNA is co-expressed in the vast majority of the cholinergic cells [35]. Thus, modulation of α7β2 nAChR heteromers offers the opportunity to selectively modulate activation of cholinergic neurons in the basal forebrain, which are important for attention and other cognitive functions [36]. 2) Nanomolar concentrations of Aβ_1–42_ are able to block activation of α7β2 nAChR heteromers, suggesting that this receptor may be uniquely affected in Alzheimer’s disease [16,20]. 3) Compared with α7 homomers, the α7β2 nAChR heteromers exhibit high sensitivity to volatile anaesthetics [37], suggesting that α7β2 nAChR heteromers in the brain may be targets for anaesthetic action.

The presence of α7β2 nAChR heteromers in the human cortex highlights the relevance of studying this receptor in relation to schizophrenia and Alzheimer’s disease. However, such studies are hampered by the lack of α7β2 nAChR selective compounds. All nAChR compounds tested so far display either unaltered or decreased current responses or EC_50_ on α7β2 heteromeric compared to α7 homomeric nAChRs [15,18,19]. It has further been shown that for ACh, the β2 subunits do not contribute to a binding site on the receptor, such that α7β2 nAChR heteromers are only activated by their α7-α7 interfaces [18]. Further efforts are necessary to reveal the impact of α7β2 nAChR heteromers on cholinergic signalling in the brain, and whether novel agonists can activate these receptors through their α7-β2 interface(s). If possible, this would offer the exciting possibility of selective activating a distinct subpopulation of nAChRs in cholinergic cells, which may be important for cognitive function, and the function of which may be hampered in Alzheimer’s disease.

In conclusion, we have demonstrated the presence of α7β2-containing heteromeric nAChRs in the human cortex. Future studies will reveal which brain regions and cell types contain these novel complexes as well as their physiological importance.
